# Nucleotide Evolution, Domestication Selection, and Genetic Relationships of Chloroplast Genomes in the Economically Important Crop Genus *Gossypium*

**DOI:** 10.3389/fpls.2022.873788

**Published:** 2022-04-15

**Authors:** Tong Zhou, Ning Wang, Yuan Wang, Xian-Liang Zhang, Bao-Guo Li, Wei Li, Jun-Ji Su, Cai-Xiang Wang, Ai Zhang, Xiong-Feng Ma, Zhong-Hu Li

**Affiliations:** ^1^Shaanxi Key Laboratory for Animal Conservation, Key Laboratory of Resource Biology and Biotechnology in Western China (Ministry of Education), College of Life Sciences, Northwest University, Xi’an, China; ^2^State Key Laboratory of Cotton Biology, Institute of Cotton Research, Chinese Academy of Agricultural Sciences, Anyang, China; ^3^Gansu Provincial Key Laboratory of Aridland Crop Science, College of Life Science and Technology, Gansu Agricultural University, Lanzhou, China

**Keywords:** cotton, domestication selection, gene flow, genetic relationship, nucleotide evolution

## Abstract

*Gossypium hirsutum* (upland cotton) is one of the most economically important crops worldwide, which has experienced the long terms of evolution and domestication process from wild species to cultivated accessions. However, nucleotide evolution, domestication selection, and the genetic relationship of cotton species remain largely to be studied. In this study, we used chloroplast genome sequences to determine the evolutionary rate, domestication selection, and genetic relationships of 72 cotton genotypes (36 cultivated cotton accessions, seven semi-wild races of *G*. *hirsutum*, and 29 wild species). Evolutionary analysis showed that the cultivated tetraploid cotton genotypes clustered into a single clade, which also formed a larger lineage with the semi-wild races. Substitution rate analysis demonstrated that the rates of nucleotide substitution and indel variation were higher for the wild species than the semi-wild and cultivated tetraploid lineages. Selection pressure analysis showed that the wild species might have experienced greater selection pressure, whereas the cultivated cotton genotypes underwent artificial and domestication selection. Population clustering analysis indicated that the cultivated cotton accessions and semi-wild races have existed the obviously genetic differentiation. The nucleotide diversity was higher in the semi-wild races compared with the cultivated genotypes. In addition, genetic introgression and gene flow occurred between the cultivated tetraploid cotton and semi-wild genotypes, but mainly *via* historical rather than contemporary gene flow. These results provide novel molecular mechanisms insights into the evolution and domestication of economically important crop cotton species.

## Introduction

Since Darwin’s time, biologists have recognized that investigating the human domestication of wild plants can help to improve our understanding of the evolutionary process (Yoo et al., 2014). Generally, domesticated forms of cultivated species differ from their wild counterparts in numerous traits (Hu et al., 2013; Mabry et al., 2021). Insights into the evolution of chloroplast genome’s domestication and selection are made possible by comparative studies of wild and domesticated representatives of individual cultivated species. In the previous study, scholars used chloroplast genome data to analyze the genetic variation and evolution of olive. As a control, the cultivated species were employed to analyze genome variation and genetic association among olive chloroplasts (Niu et al., 2020). Meanwhile, some other study have also examined the evolutionary mechanism of the chloroplast genome of cultivated *Camellia sinensis* and its relatives (Li et al., 2021). In recent studies, comparisons of wild and domesticated plants have provided important insights into the developmental mechanisms that underlie traits affected strongly due to targeted selection by humans (Yoo et al., 2014). In general, domesticated plants are characterized by reduced genetic variation and relaxed selection pressure compared with their wild counterparts. Several studies also found high levels of continuous gene flow from wild to cultivated genotypes (Price, 2002; Burger et al., 2008; Gross and Olsen, 2010; Ma et al., 2019). Thus, the domestication process may provide a basis for studying the overall evolutionary relationships associated with wild crop transformation and identifying the genes under selection (Gepts, 2004; Burger et al., 2008).

Cotton (*Gossypium*) is one of the most important crops worldwide (Wendel, 1989; Ruan, 2003) and a major source of natural fiber for the textile industry. Allopolyploid cotton originated in the New World and diverged into at least six species throughout the tropical and subtropical Americas: *G. hirsutum* (AD_1_), *G. barbadense* (AD_2_), *G. tomentosum* Nuttalex Seemann (AD_3_), *G. mustelinum* Miersex Watt (AD_4_), *G*. *darwinii* Watt (AD_5_), and *G. ekmanianum* (AD_6_) (Wendel and Cronn, 2003; Wendel and Grover, 2015). The diploid species comprise eight monophyletic genome groups: A, B, C, D, E, F, G, and K (Wendel and Cronn, 2003; Grover et al., 2007; Wendel et al., 2010). These groups can be separated into three main lineages in three continental regions: 13 D-genome species from the American continents, 15 species from the Asian and African continents (A-, B-, E-, and F-genomes), and 18 species (C-, G- and K-genomes) from Australia (Wendel and Cronn, 2003). Hence, cotton species provide a fascinating model system for studying evolution, domestication selection, genetic introgression, and gene flow among different continents (Fryxell, 1969, 1978; Wendel, 1989; Wendel and Grover, 2015; Chen et al., 2016, 2017a,2017b). Four species in the genus *Gossypium* are cultivated for the production of spinnable fiber, i.e., two allotetraploid species comprising *G*. *hirsutum* L. and *G*. *barbadense* L. (2n = 4x = 52), and two diploid species comprising *Gossypium herbaceum* L. (A_1_) and *Gossypium arboretum* L. (A_2_) (2n = 2x = 26) (Wendel and Cronn, 2003; Wendel and Grover, 2015). Allopolyploid cottons were considered to be about 1.5 million years old and were domesticated by humans 4,000 to 5,000 years ago (Wendel, 1989; Wang et al., 2017), which were originally domesticated from tree cotton in the Mesoamerican and Caribbean regions, and then further domesticated and improved in the southern United States (Fang et al., 2017). And two diploid cotton species, *G*. *arboretum* and *G*. *herbaceum*, have been cultivated for several millennia (Simon et al., 2016), which were initially domesticated on Madagascar or in the Indus Valley (Mohenjo Daro), and was subsequently dispersed to Africa and other areas of Asia (Wendel and Grover, 2015; Du et al., 2018; Huang et al., 2020). Due to the high-yield characteristics of allopolyploid cottons, the American upland cottons have been introduced and replaced by two diploid cotton species (*G*. *arboretum* and *G*. *herbaceum*) (Fang et al., 2017; Du et al., 2018). Up to now, the Upland cotton (*G. hirsutum*) accounts for more than 95% of the worldwide production of cotton (Yoo et al., 2014; Fang et al., 2017; Ma et al., 2019; Wang et al., 2019; Zhang et al., 2020).

Following human-mediated selection and agronomic improvement, the ability of cotton species to adapt to various environments was enhanced and the production of fiber from cotton improved significantly (Ma et al., 2019). The domestication process also resulted in other morphological changes in other crops such as sorghum, rice and soybean (Ma et al., 2019), including early flowering, larger and/or more fruits, annualized habit, plant height reduction, and loss of seed dormancy (Yoo et al., 2014). When plants undergo artificial domestication, the relaxation of certain features is inevitable (Price, 2002), that is, when plants undergo relatively large changes, such as from the transition from nature to domestication, certain characteristics important for survival in nature lose much of their adaptive significance under artificial directional selection. Hence, one would expect natural selection for such characteristics to lose its intensity (Coss, 1999; Price, 2002). Many studies have shown that the genetic diversity of upland cotton varieties is low, mainly due to several bottlenecks in the domestication process (Brubaker and Wendel, 1994; May et al., 1995; Iqbal et al., 2001; Wendel and Cronn, 2003). In addition, previous studies based on whole-genome resequencing of upland cotton have indicated that the genomic diversity of upland cotton decreased under the stress of artificial selection (Fang et al., 2017; Ma et al., 2019). Thus, in the current era of genomic big data, high-throughput “omics” sequencing techniques allow detailed analyses of the genetic changes associated with artificial domestication, as well as providing new, accurate, and targeted genome-based crop breeding strategies (Wang et al., 2017; Li et al., 2020; Yang et al., 2020). For example, in maize and rice, the use of high-quality backbone parents can obtain notable improvements in breeding efficiency (Ma et al., 2019). The whole-genome sequences of allotetraploid cotton and its ancestors have been completed, and the high-quality allotetraploid upland cotton genome is an effective tool for systematically exploring the genomic mysteries of polyploidy (Li et al., 2014, 2015; Zhang et al., 2015). Compared with whole-genome sequencing, the chloroplast genome is single-copy, maternally inherited, and there is no chain exchange or free combination phenomenon. It has a relatively independent evolutionary route. In addition, the highly conserved characteristics of the chloroplast genome make them useful for the rapid analysis of species evolution (Jansen et al., 2007; Parks et al., 2009; Wang et al., 2013; Chen et al., 2014). However, the whole-genome resequencing (WGR) is parental inheritance, and there may be genetic recombination (Gover et al., 2020; Wang et al., 2020).

In the current study, to better understand the evolution, domestication selection, and genetic relationships of cotton, we analyzed the chloroplast genomic variation in 72 cotton genotypes comprising *G. hirsutum* and its 29 cultivated upland cotton accessions, *G*. *barbadense* and its three cultivated accessions (*Gossypium barbadense* cultivar zhonghai 7, *Gossypium barbadense* cultivar Kaiyuan, and *Gossypium barbadense* cultivar yuanmou), *G*. *africanum*, *G*. *arboretum*, seven semi-wild races of *G*. *hirsutum*, and 29 wild cotton species. We also estimated molecular dating, genetic introgression, nucleotide substitutions, and indel variation.

## Materials and Methods

### DNA Extraction and Plant Materials

The fresh leaves of seven semi-wild races of upland cotton, i.e., punctatum, latifolium, richmondi, morrilli, marie-galante, palmeri, and yucatanense, were collected from the National Wild Cotton Nursery in Sanya, China. In addition, 29 cultivated upland cotton accessions were also obtained from different ecological geographic regions, with three accessions from the United States, eight from the Yellow River region, 12 from the Yangtze River area, four from northwest China, and two from north China (Table 1). Leaf tissues were dried with silica gel and genomic DNA was extracted using the modified CTAB method (Doyle and Doyle, 1987). Approximately 5 μg of purified DNA was used to construct paired-end libraries with an insert size of 350 bp and sequencing was performed with the Illumina HiSeq 2500 platform by Novogene (Beijing, China). Additionally, we have also downloaded the 36 chloroplast genomes of cotton species from NCBI (National Center for Biotechnology Information) for further combination analysis.

**TABLE 1:** List of taxa sampled in this study and species accession numbers (GenBank).

Number	Species	Accession number	Source	Logogram
1	*Gossypium punctatum*	MK792868	Sanya, China	JBM
2	*Gossypium richmondii*	MK792869	Sanya, China	lqmd
3	*Gossypium morrilli*	MK792866	Sanya, China	MLE
4	*Gossypium marie-galante*	MK792865	Sanya, China	MLJ
5	*Gossypium palmerii*	MK792867	Sanya, China	PME
6	*Gossypium yucatanense*	MK792870	Sanya, China	YKT1
7	*Gossypium hirsutum* cultivar 06G415	MK792871	Yellow river	S32
8	*Gossypium hirsutum* cultivar antongSP21	MK792837	United States	S24
9	*Gossypium hirsutum* cultivar chuanmian45	MK792838	Yangtze river	S47
10	*Gossypium hirsutum* cultivar CJL-233	MK792839	Yangtze river	S252
11	*Gossypium hirsutum* cultivar difenmian168	MK792840	Yangtze river	S64
12	*Gossypium hirsutum* cultivar ekangmian7	MK792841	Yangtze river	S273
13	*Gossypium hirsutum* cultivar emian12(4947)	MK792842	Yangtze river	S263
14	*Gossypium hirsutum* cultivar gaochanbukangchong RRM	MK792843	Yangtze river	S246
15	*Gossypium hirsutum* cultivar guangyedaizimian	MK792844	United States	S59
16	*Gossypium hirsutum* cultivar guokang12 (GK12)	MK792845	Yellow river	S156
17	*Gossypium hirsutum* cultivar hanmian802	MK792846	Yellow river	S162
18	*Gossypium hirsutum* cultivar humian204	MK792847	Yangtze river	S257
19	*Gossypium hirsutum* cultivar Jan-86	MK792848	Yellow river	S211
20	*Gossypium hirsutum* cultivar liaomian10	MK792849	North China	S234
21	*Gossypium hirsutum* cultivar lumianyan21(lu1138)	MK792850	Yellow river	S163
22	*Gossypium hirsutum* cultivar shan401	MK792851	Yellow river	S10
23	*Gossypium hirsutum* cultivar simian4	MK,792852	Yangtze river	S272
24	*Gossypium hirsutum* cultivar sizimian4	MK792853	United States	S38
25	*Gossypium hirsutum* cultivar sumian5	MK792854	Yangtze river	S45
26	*Gossypium hirsutum* cultivar xinluzhong7	MK792855	Northwest China	S275
27	*Gossypium hirsutum* cultivar xinluzhong9 (1318136-160)	MK792856	Northwest China	S277
28	*Gossypium hirsutum* cultivar xinluzhong10	MK792857	Northwest China	S278
29	*Gossypium hirsutum* cultivar xinluzhong19	MK792858	Northwest China	S281
30	*Gossypium hirsutum* cultivar xuzhou209	MK792859	Yangtze river	S13
31	*Gossypium hirsutum* cultivar yanmian48	MK792860	Yangtze river	S265
32	*Gossypium hirsutum* cultivar youLU272⊕	MK792861	Yellow river	S175
33	*Gossypium hirsutum* cultivar yumian1	MK792862	Yangtze river	S271
34	*Gossypium hirsutum* cultivar zhong053	MK792863	Yangtze river	S8
35	*Gossypium hirsutum* cultivar zhongzhimian GD89	MK792864	Yellow river	S185
36	*Gossypium barbadense* cultivar zhonghai7	HQ901199	NCBI	AD_2_ 99_
37	*Gossypium barbadense* cultivar kaiyuan	HQ901200	NCBI	AD_2_200_
38	*Gossypium barbadense* cultivar yuanmou	HQ901198	NCBI	AD_2_98_
39	*Gossypium darwinii*	NC_016670	NCBI	AD_5_70_
40	*Gossypium tomentosum*	NC_016690	NCBI	AD_3_90_
41	*Gossypium mustelinum*	NC_016711	NCBI	AD_4_
42	*Gossypium hirsutum*	NC_007944	NCBI	AD_1_44_
43	*Gossypium barbadense*	NC_008641	NCBI	AD_2_41_
44	*Gossypium africanum*	NC_016692	NCBI	A_1_*a*_
45	*Gossypium arboreum*	NC_016712	NCBI	A_2_
46	*Gossypium longicalyx*	JF317354	NCBI	F_1_
47	*Gossypium anomalum*	JF317356	NCBI	B_1_
48	*Gossypium capitis-viridis*	NC_018111	NCBI	B_3_
49	*Gossypium sturtianum*	JF317353	NCBI	C_1_
50	*Gossypium nandewarense*	MG779276	Sanya, Hainan, China	C_1–n_
51	*Gossypium robinsonii*	NC_018113	NCBI	C_2_
52	*Gossypium bickii*	JF317352	NCBI	G_1_
53	*Gossypium australe*	NC_033401	NCBI	G_2_
54	*Gossypium populifolium*	NC_033398	NCBI	K_2_
55	*Gossypium thurberi*	JF317353	NCBI	D_1_
56	*Gossypium armourianum*	MG891801	Sanya, Hainan, China	D_2–1_
57	*Gossypium harknessii*	NC_033333	NCBI	D_2–2_
58	*Gossypium klotzschianum*	NC_033394	NCBI	D_3–k_
59	*Gossypium davidsonii*	NC_033395	NCBI	D_3–d_
60	*Gossypium aridum*	NC_033396	NCBI	D_4_
61	*Gossypium raimondii*	NC_016668	NCBI	D_5_
62	*Gossypium gossypioides*	NC_017894	NCBI	D_6_
63	*Gossypium lobatum*	MG891802	Sanya, Hainan, China	D_7_
64	*Gossypium trilobum*	MG800783	Sanya, Hainan, China	D_8_
65	*Gossypium laxum*	KF806549	NCBI	D_9_
66	*Gossypium turneri*	NC_026835	NCBI	D_10_
67	*Gossypium schwendimanii*	MG891803	Sanya, Hainan, China	D_11_
68	*Gossypium stooksii*	JF317354	NCBI	E_1_
69	*Gossypium somalense*	NC_018110	NCBI	E_2_
70	*Gossypium areyiabum*	NC_018112	NCBI	E_3_
71	*Gossypium incanum*	NC_018109	NCBI	E_4_
72	*Gossypium latifolium*	MG800784	Sanya, Hainan, China	kym

### Chloroplast Genome Assembly, and Annotation

The raw sequencing reads obtained by the company (Novogene, Beijing, China) were filtered through the “AmbiguityFiltering.pl” script in the NGSQCToolkit software (Patel and Jain, 2012), and removed the fragments with fuzzy bases greater than 2% and those with bases less than 50 bp. The clean reads were assembled by the MIRA 4.0.2 program (Chevreux et al., 2004) where the complete chloroplast genome of *G. hirsutum* (AD_1_) (NC_007944) was used as the reference sequence in this process. In order to further assemble the whole chloroplast genomes, some ambiguous regions were extended using the MITObim v1.7 program with a baiting and iteration method (Hahn et al., 2013). The contigs obtained were used to generate consensus sequences with Geneious v8.0.2 (Kearse et al., 2012). The chloroplast genomes were then annotated using the Dual Organellar Genome Annotator (DOGMA, Wyman et al., 2004) program and manual corrections were made for some specific genes. All tRNA genes were further confirmed using the online tool tRNAscan-SE (Schattner et al., 2005). All of the newly generated genome sequences were submitted to GenBank (accession numbers MK792837–MK792871 and MG800784).

### Genetic Clustering Analysis

To evaluate the genetic relationships among cotton genotypes, molecular phylogenetic analysis was conducted using 72 complete chloroplast genome sequences (Table 1) and two outgroups comprising *Bombax ceiba* (NC_037494) and *Theobroma cacao* (NC_014676). First, all of the sequences were aligned using the MAFFT program (Katoh and Standley, 2013) and the best-fit model was then selected with Modeltest v3.7 (Posada and Crandall, 1998) based on Akaike’s information criterion. Finally, a maximum likelihood tree was constructed using RAxML v7.2.8 (Stamatakis, 2006) where the best model was GTR + G based on 1000 bootstrap replicate tests.

### Estimation of Divergence Times

Previously estimated dates of speciation events (fossil records) were used to calibrate the phylogenetic tree (Pfeil and Crisp, 2008). In BEAST v1.8.0 (Drummond et al., 2012), we used the Yule process speciation prior and the uncorrelated lognormal model of rate change with a relaxed clock to estimate the divergence times among cotton lineages. The divergence time was calculated based on 74 chloroplast protein-coding sequences shared by the cotton genotypes, and we used three fossil records: AD_1_ (*G*. *hirsutum*) and A_2_ (*G*. *arboreum*) diverged 1–2 Mya (Wendel, 1989), A_2_ (*G*. *arboreum*) and D_5_ (*G*. *raimondii*) diverged ∼ 5–10 Mya (Senchina et al., 2003), and *Theobroma*-*Gossypium* diverged 60 Mya (Carvalho et al., 2011). A normal prior probability distribution was used to account for the uncertainty of prior knowledge. The analyses were run for 50,000,000 generations and the parameters were sampled every 5,000 generations. Tracer v 1.6 (Drummond et al., 2012) was used to determine the effective sample size (>200) and the first 20% of the samples were discarded as burn-in. Tree Annotator v.1.8.0 (Drummond et al., 2012) was used to summarize the set of post-burn-in trees and their parameters were used to produce a maximum clade credibility chronogram, which illustrated the mean divergence time estimates in the 95% highest posterior density (HPD) intervals. Finally, FigTree V1.3.1 (Drummond et al., 2012) was used to visualize the molecular dating estimates.

### Analysis of Nucleotide Substitutions

Transitions/transversions explain the substitution rates of nucleotides, so we determined the transition/transversion rates using single nucleotide polymorphism (SNP) loci in protein-coding sequences in the cotton chloroplast genome. These analyses were conducted based on two genetic groups obtained from the phylogenetic analyses. One group contained the diploid cotton species (including *G*. *africanum* and *G*. *arboretum*) and the other group comprised tetraploid semi-wild races and cultivated upland cotton genotypes (excluding *G*. *barbadense*). MEGA files generated from SNP data were analyzed with MEGA7 software (Kumar et al., 2016) to obtain the transition/transversion rate. The following parameters were employed: statistical method, maximum likelihood; analysis, substitution pattern estimation (MCL); substitution type, nucleotides; scope, all selected taxa; model/method, Tamura–Nei (automatic selection); gaps/missing data treatment, partial deletion, and site coverage cut off (%), 95 (Mohanta and Bae, 2017). Finally, we converted the transition/transversion rates for the two groups into two histograms. In addition, DnaSP v5.10 (Librado and Rozas, 2009) was used to calculate the non-synonymous (dN) and synonymous (dS) mutations in coding regions for the two groups.

### Estimation of Mutation Rates

The two cotton groups described above were also used to calculate the mutation rates. The rate of mutation per site per year (μ) was estimated using the formula: μ = *m*/(*nT*), where *m* is the number of observed mutations, *n* is the number of total sites, and *T* is the divergence time of a node (Denver et al., 2009). The μ values for structural mutations were calculated using the method described by Saiton and Ueda (Saitou and Ueda, 1994), where the total number of structural mutations was divided by the additive time based on the branch lengths and by the length of the nucleotide sequences. Finally, we calculated the evolutionary rates for nucleotide substitutions and indels. The indel rates were calculated for the two groups using DnaSP v5.10 (Librado and Rozas, 2009).

### Selection Pressure Analysis

To identify domestication selected genes, we performed selection pressure analysis using the Codeml program (Yang et al., 2005) and two different groups of genotypes, where one group comprised the wild diploid cotton species with a total of 28 genotypes and the other group contained the upland cotton semi-wild races and cultivated varieties with a total of 37 cotton genotypes (excluding *G*. *barbadense* and its three cultivated accessions, i.e., *G*. *tomentosum*, *G*. *mustelinum* and *G*. *darwinii*, because these seven genotypes were not involved in the domestication selection process for upland cotton). In general, the non-synonymous (dN) and synonymous substitution (dS) rate ratio (ω = dN/dS) was sensitive to selection pressure during evolution at the protein level, and it was particularly useful for identifying positive selection. Geneious v8.0.2 (Kearse et al., 2012) and MAFFT v7.0.0 (Katoh and Standley, 2013) were used to extract and align 77 protein-coding chloroplast genes from the two groups. Maximum likelihood phylogenetic trees were constructed based on the complete chloroplast genome sequences using RAxML v7.2.8 (Stamatakis, 2006). This model allowed the ω ratio to vary among sites with a fixed ω ratio for the whole tree to test for site-specific evolution in the gene phylogeny (Yang and Nielsen, 2002). Log-likelihood values of every model were compared against a neutral model based on likelihood ratio tests in order to determine statistically significant differences. Only the candidate sites for positive selection with significant support based on the posterior probability (*p* of (ω > 1) ≥0.99; Bayes Empirical Bayes approach) identified by M2 and M8 were considered further.

### Diversity and Genetic Structure Analysis

DnaSP v5.10 (Librado and Rozas, 2009) was used to analyze the genetic diversity parameters based on the complete chloroplast genome sequences of seven semi-wild races and 29 cultivated upland cotton genotypes. We also calculated the haplotype diversity (*H*_d_) (Nei and Tajima, 1981), nucleotide diversity (π) (Nei and Li, 1979), and the number of haplotypes (*H*) with DnaSP v5.10 software.

We also analyzed the genetic structure patterns using the Bayesian Markov chain Monte Carlo clustering analysis method implemented in STRUCTURE 2.3.3 (Pritchard et al., 2000; Falush et al., 2003; Hubisz et al., 2009). The admixture model with correlated allele frequencies was implemented for each run without a prior placed on the population information (Hubisz et al., 2009). We conducted eight independent runs for each value from *K* = 1–10 to estimate the “true” number of clusters in 200,000 Markov chain Monte Carlo cycles following a burn-in step of 500,000 iterations. The most likely number of clusters was defined using log probabilities [Pr(*X*| *K*)] (Pritchard et al., 2000) and the △*K* method (Evanno et al., 2005) *via* the online website STRUCTURE HARVESTER (Earl and VonHoldt, 2012). Next, CLUMPP 1.1.2 and the Greedy algorithm were used to align multiple runs of STRUCTURE for the same *K* value (Jakobsson and Rosenberg, 2007). Finally, we applied DISTRUCT 1.1 (Rosenberg, 2004) to graphically visualize the individual probabilities of cluster membership.

### Gene Flow

We calculated the historical gene flow in semi-wild races and cultivated upland genotypes using Migrate-n (Beerli, 2006). First, we generated five independent Markov chain Monte Carlo cycles, each with 5,000,000 generations. We then sampled every 100 steps under a constant variation model and discarded the first 1,000,000 records as a burn-in and the other settings were at their default values. After checking for data convergence, we estimated the mode and 95% HPD (Du et al., 2017). In addition, we applied BAYESASS v3.0 to detect contemporary gene flow in the two groups (Wilson and Rannala, 2003). In these calculations, the three parameters comprising the migration rates (Δ*M*), allele frequencies (Δ*A*), and inbreeding coefficients (Δ*F*) were used as references to ensure that the optimal acceptance rates for the three parameters fell within the range of 20–60%. After continuous calculations, the correlation values for the genetic components were finally determined as 0.03, 0.16, and 0.14, respectively. We then conducted the analyses based on 5^7^ iterations after a burn-in of 5^6^ iterations and set 1,000 as the sampling frequency. Ten separate runs were performed to minimize the convergence problem (Feng et al., 2016). The method proposed by Meirmans was used to obtain the results with the lowest deviance (Meirmans, 2014).

## Results

### Evolutionary Relationships

**FIGURE 1:**
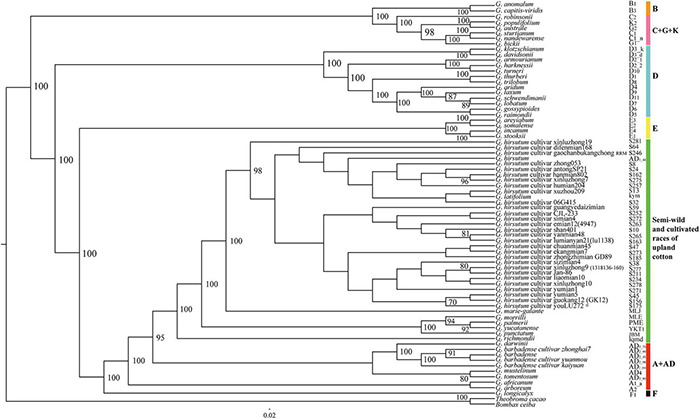
Phylogenetic relationships among 72 Gossypium accessions based on complete chloroplast genomes. Green represents the cultivated accessions and semi-wild races of upland cotton, and other colors represent six genetic clades. *B. ceiba* and *T. cacao* were used as outgroups.

The chloroplast genome sequences and concatenated protein-coding genes were used to reconstruct the maximum likelihood phylogenetic relationships for 72 *Gossypium* genotypes, and the cotton relationships generated from the data sets had the same topology, as shown in Figure 1. The six major genetic clades identified comprised the A + AD, F, E, D, B, and C + G + K genomic groups. Interestingly, all of the cultivated upland cotton genotypes clustered with the semi-wild race latifolium, which also formed a large evolutionary lineage with the other semi-wild races. The A-genome cotton species and *G*. *barbadense* genotypes also formed a single clade and they were closest to the upland cotton branch, whereas the 13 D-genome species formed a strong monophyletic lineage. The Australian species (C + G + K) clustered into a small branch, which clustered into a large branch with the B-genome species. Four species representing the E-genomic group also clustered into a large evolutionary branch. These results were in good agreement with the biogeographic distributions of cotton species from different continents.

### Divergence Time Estimation

**FIGURE 2:**
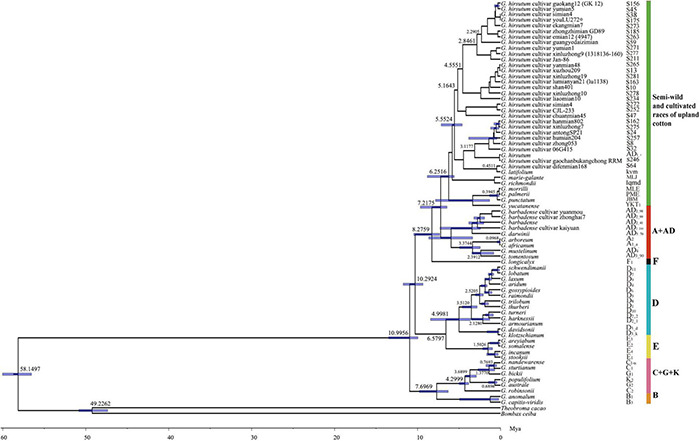
Divergence time tree obtained for cotton accessions based on 72 chloroplast protein-coding sequences.

The molecular dating showed that the divergence time between the genus *Gossypium* and outgroups (*B. ceiba* and *T. cacao)* was about 58.15 Mya (95% HPD = 56.53–60.04 Mya), which are consistent with previous estimates (Carvalho et al., 2011; Figure 2). The genus *Gossypium* originated about 11 Mya (95% HPD = 9.34–11.74 Mya) and most genomic groups in the genus diverged radially in a relatively narrow time range. Interestingly, the divergence time between the B-genome (African origin) and Australian clades (C + G + K) was estimated at 7.7 Mya (95% HPD = 6.3–9.8 Mya), which again supported the genetic relationship present in the B-genome, i.e., the B-genome branch and Australian branch were strongly grouped phylogenetically. The semi-wild races and cultivated upland cotton accessions diverged about 3.12 Mya and the ancestor of the D-genome originated at 5 Mya (95% HPD = 3.59–5.44 Mya). The divergence time of the allotetraploid AD clade was about 3.37 Mya (95% HPD = 2.44–4.93 Mya).

### Nucleotide Substitutions

**FIGURE 3:**
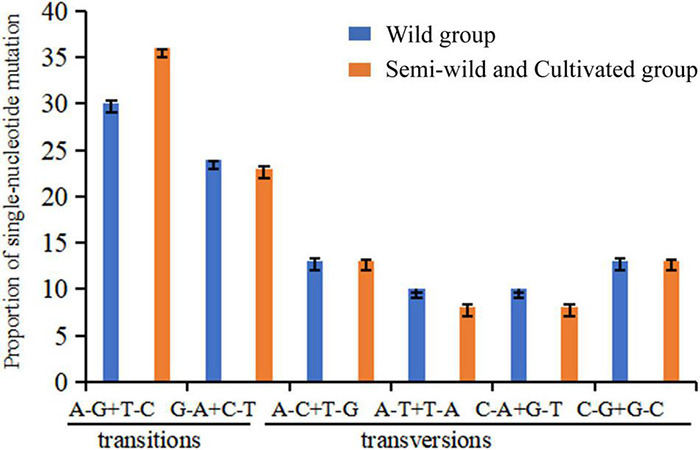
Nucleotide substitution patterns in wild cotton species, semi-wild races, and cultivated cotton accessions based on SNP site variations. The patterns were divided into six types as indicated by the six non-strand-specific base substitution types. *p* = 0.97681. Because the calculated value is a fixed value with a decimal. We calculate the error bar between the actual value and the integer substitution site. The *p*-value is calculated by *T*-test.

The ratios of transition/transversion were high among the semi-wild races and cultivated upland cotton genotypes (1.41), but low among the genotypes of the wild cotton species (1.16) (Table 2). There were significant differences in the proportions of two transition mutations and four transversion mutations between the two groups (Figure 3). Among the four transversion mutations, the proportion of A-C + T-G mutations was similar to that of C-G + G-C mutations in the groups. In addition, few A-T + T-A and C-A + G-T mutations were found in all combinations.

**TABLE 2:** Ratios of transitions and transversions for plastid protein-coding sequences in cotton accessions.

	Semi-wild and cultivated cotton accessions					Wild cotton species				
From\To	A	T	C	G	Ts/Tv	A	T	C	G	Ts/Tv
A	–	4.2727	4.2655	**22.2241**	1.4100	–	4.6792	6.4654	**13.9443**	1.1600
T	3.3880	–	**13.9884**	8.5670		5.7307	–	**15.6745**	6.2325	
C	3.3880	**14.0120**	–	8.5670		5.7307	**11.3441**	–	6.2325	
G	**8.7889**	4.2727	4.2655	–		**12.8216**	4.6792	6.4654	–	

*Rates of different transitional substitutions are shown in bold, whereas those of transversional substitutions are not shown in bold.*

The 4,074 biallelic SNPs were subdivided into coding, intron, and intergenic spacer regions, and sorted into two groups comprising wild cotton species, and semi-wild and cultivated upland cotton genotypes (Table 3). In wild cotton group, there were 3,753 SNPs in total: 1,375 in coding regions, 264 in intron regions, and 2,693 in intergenic spacer regions. The percentages of SNP to the total lengths were 1.72, 1.22, 2.95, respectively, manifesting the intergenic spacer region sequences were more variable than the intron regions. In the coding regions, there were 1,027 non-synonymous mutations and 347 synonymous mutations, and the dN/dS was about 2.96. In the semi-wild and cultivated cotton genotypes, the sequences of the intergenic spacers and intron regions were more variable than the coding regions. The dN/dS ratio (3.5) was larger for this group than the wild cotton species (56 non-synonymous mutations and 16 synonymous mutations).

**TABLE 3:** Taxonomic and genomic distribution of biallelic single nucleotide polymorphic loci in wild, semi-wild, and cultivated cotton plastid genomes.

		Wild accessions			Semi-wild and cultivated accessions	
Genome region	Length (bp)	Value	%	Length (bp)	Value	%
Total substitutions	163,400	3,753	2.3	166,237	321	0.19
Coding region	79,704	1,375	1.72	79,968	77	0.1
Non-synonymous	/	1,027	1.29	/	56	0.07
Synonymous	/	347	0.44	/	16	0.02
dN/dS	/	2.96	/	/	3.5	/
Intron	21,581	264	1.22	21,292	14	0.07
Intergenic spacer	81,381	2,693	2.95	77,524	130	0.17

### Estimation of Mutation Rate

The evolutionary rates were calculated based on the lengths of the genomes, number of substitutions, and times since divergence. In total, 1,375 substitutions were estimated in the wild species group and 77 in the semi-wild races and cultivated upland cotton group. The evolutionary rate of nucleotide substitutions was 1.2 × 10^–9^ per site per year in the wild species group compared with 0.18 × 10^–9^ per site per year in the semi-wild and cultivated group. In addition, 479 indels were identified in the wild cotton species and the evolutionary rate for indels was estimated at 0.4 × 10^–9^ per site per year. In the semi-wild and cultivated group, 24 indels were detected and the evolutionary rate was estimated at 0.05 × 10^–11^ per site per year.

### Selection Pressures

We identified 16 genes with sites under positive selection in the wild species group (Supplementary Tables 1, 2). These genes comprised two ATP subunit genes (*atpB* and *atpE*), three ribosome small subunit genes (*rps2*, *rps3*, and *rps12*), three genes encoding cytochrome b/f complex subunit proteins (*petB*, *petD*, and *petN*), one NADH oxidoreductase gene (*ndhG*), one DNA-dependent RNA polymerase gene (*rpoC2*), one gene encoding ribosome large subunit protein (*rpl16*), and five other genes (*ccsA*, *cemA*, *rbcL*, *ycf1*, and *ycf2*). According to the M2 and M8 models, the *rps12* gene harbored 28 sites under positive selection, as well as 34 sites in *ycf2*, six and four sites in *ycf1*, two and five sites in *ndhG*, and one site each in the *ccsA*, *cemA*, *rpl16*, *rps3*, and *petB* genes. The M8 model detected 15 sites under positive selection in the *rps2* gene. However, sites under positive selection in the *atpB* (five), *atpE* (two), and *rbcL* (two) genes were only detected by the M2 model, and the other six genes had only one active site.

We only identified the ribosome large subunit protein (*rpl2*) gene with sites under positive selection in the semi-wild and cultivated group, where it harbored four sites under positive selection in the M2 model (Supplementary Tables 3, 4).

### Diversity and Genetic structure

**FIGURE 4:**
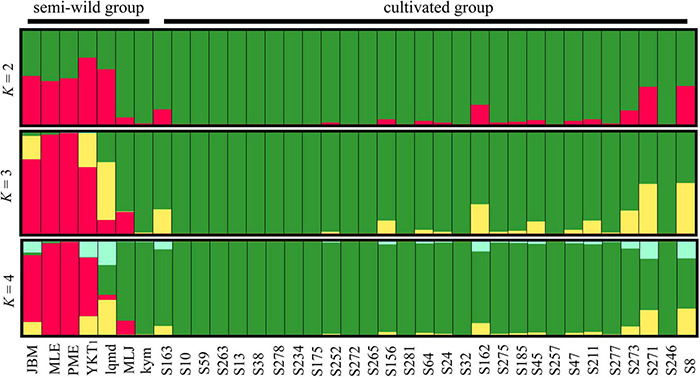
Bayesian clustering results obtained for cotton accessions by STRUCTURE analysis. Each individual is represented by a vertical bar, which is partitioned into K colored segments showing the individual’s probability of belonging to the group with that color.

Seven chloroplast DNA haplotypes were identified in the semi-wild races and 22 in the cultivated upland cotton genotypes (Table 4). The haplotypes diversity (*H*_d_) and π values were slightly higher for the semi-wild races than the cultivated genotypes. STRUCTURE analyses and the Δ*K* statistic indicated an “optimal” value for *K* (number of populations modeled) of 2 (Supplementary Figure 1), thereby supporting the existence of two major clusters in the data set (Figure 4). The semi-wild races were primarily assigned to cluster I and the cultivated genotypes to cluster II, whereas the races marie-galante and latifolium had notable fractions assigned to cluster II, thereby suggesting genetic introgression between the two groups.

**TABLE 4:** Nucleotide diversity and haplotype frequencies for plastid genomes in semi-wild and cultivated accessions of upland cotton.

Population	Number of samples	Number of haplotypes (H)	Hd (SD)	π (SD) × 100	Number of segregation sites	Theta
Semi-wild races	7	7	1.000 (0.076)	0.00035 (0.00006)	157	0.196
Cultivated accessions	29	22	0.946 (0.035)	0.00010 (0.00003)	170	0.132

### Gene Flow

Patterns of historical and contemporary gene flow were detected between the semi-wild and cultivated upland cotton genotypes. Migrate-n analysis showed that historical gene flow ranged from 149.77 (135.69–164.85) for the semi-wild group to 377.47 (344.25–413.03) for the cultivated group, thereby indicating asymmetric gene flow between the groups. Significant asymmetric contemporary gene flow was also found between the groups, where the values ranged from 0.1110 (0.0612–0.1608) for the semi-wild group to 0.0108 (0.0004–0.0212) for the cultivated group. These results suggest a higher level of historical gene flow during domestication compared with the low level of contemporary gene flow.

## Discussion

### Evolutionary Relationships

Some previous studies have explored the molecular phylogenetic relationships of cotton, mostly based on a small number of plastids and nuclear DNA markers, as well as the complete chloroplast genome sequence and mitochondrial genome data set of a limited number of cotton species (Cronn et al., 2002; Senchina et al., 2003; Wendel et al., 2009; Xu et al., 2012; Wendel and Grover, 2015; Chen et al., 2016, 2017a,2017b; Wu et al., 2018). However, the relationship between cultivated accessions of upland cotton and other species of *Gossypium* is not clear now. Therefore, we built phylogenetic analyses on 72 cotton plastid genome sequences including wild species, semi-wild races and cultivated accessions of *Gossypium*, representing the largest number of known cotton species. In the phylogenetic tree, *Gossypium* species were primarily divided into three large genetic branches. The outer two branches mainly comprised diploid cotton species and the upland cotton clade formed the inner branch. One of the two outside branches included the Australian species with C, G, and K-genomes, American D-genome species, and African E- and B-genome species. Other studies have also shown that species with the G-genome have a common nested relationship with C-genome species, probably due to the frequent capture of chloroplasts in the *G*. *bickii* lineage (Seelanan et al., 1999; Liu et al., 2001). The other outside branch comprised the African F-genome species, Asian–African A-genome species, and American AD-genome wild species and cultivated *G*. *barbadense* genotypes. The large internal branch included all of the upland cotton cultivars and semi-wild races. The race latifolium clustered more closely with the upland cotton genotypes, which may suggest a classification error because the race yucatanense is considered the closest progenitor of cultivated upland cotton. Some studies have reported that the maternal donor of the chloroplast genome for the allotetraploid species was the A-genome progenitor (Cronn et al., 2002; Chen et al., 2016, 2017a; Huang et al., 2020), and this was supported by our phylogenetic analysis. The latest research showed that the two A-genome species (*G*. *herbaceum* and *G*. *arboreum*) have evolved independently with no ancestor-progeny relationship (Huang et al., 2020). In addition, the phylogenetic tree showed that all 13 D-genome species clustered into a single lineage with high support and they were more distantly related to the upland cotton genotypes. Some D-genome species formed closely associated pairs, including *G*. *klotzschianum* (D_3–k_) with *G*. *davidsonii* (D_3–d_), *G*. *harknessii* (D_2–2_) with *G*. *turneri* (D_10_), *G*. *thurberi* (D_1_) with *G*. *trilobum* (D_8_), and *G*. *raimondii* (D_5_) with *G*. *gossypioides* (D_6_). These results are consistent with previous reports of phylogenetic relationships based on nuclear genetic markers and chloroplast genome sequences (Alvarez et al., 2005; Ulloa et al., 2013; Chen et al., 2017a; Wu et al., 2018; Huang et al., 2020). The difference in phylogenetic relationships may be caused by the different genetic characteristics of the DNA markers used.

### Divergence Time Analysis

We estimated the divergence time of *Gossypium* species based on the plastid protein-coding sequences. The results showed that the diversification between *Gossypium* and *T*. *cacao* was found to have occurred about 58 Mya, which was consistent with previous inferred results (Wendel, 1989; Senchina et al., 2003; Carvalho et al., 2011; Chen et al., 2016). Interestingly, the divergence time was estimated at 7.7 Mya (95% HPD = 6.3–9.8 Mya) between the B-genome and Australian clade (C + G + K), which was similar to the rapid radiation time calculated for all other cotton branches after differentiation from Australian cotton species (Chen et al., 2016). In addition, the evolutionary time of the cotton ancestors was 11 Mya and cotton species then rapidly differentiated radially, where the differentiation time of most branches was 5–6 Mya. These results were largely consistent with those obtained in other molecular studies (Chen et al., 2016, 2017a,2017b). The differentiation time for the semi-wild races, cultivated upland cotton genotypes, and AD-genome was estimated at 6.25 Mya, and that estimated for the race latifoloum and *Gossypium hirsutum* cultivar difenmian168 was 0.45 Mya. We also found that the divergence time betweem semi-wild races and cultivated upland cotton accessions were about 3.12 Mya, thereby indicating that they may have differentiated recently. The evolutionary time for the allotetraploid upland cotton accessions was 6.25 Mya (6.4–9.7), which agrees with the results obtained in previous studies (Senchina et al., 2003; Wang et al., 2017; Ma et al., 2019; Huang et al., 2020), where it was domesticated at least 4,000 to 5,000 years ago and subsequently subjected to direct selection (Wang et al., 2017). To the best of our knowledge, the present study is the first to use the protein-coding sequences in the chloroplast genome to estimate the divergence dates of the whole *Gossypium* species including semi-wild races and cultivated upland cotton genotypes, although the results could be improved by larger phylogenetic analyses.

### Genetic Mutation

Mutation is the ultimate source of genetic variation, the substrate of evolution (Nachman and Crowell, 2000; Zhang et al., 2020). A previous study suggested that the mutation/substitution rates varied between and within genomes (Mohanta and Bae, 2017), and that they were influenced by factors such as the nearest neighbor bases, chromosomal position, and the efficiency of the repair systems between the leading and lagging DNA strands. In general, the presence of similar bases or derivatives of similar bases facilitates the base replacement in the DNA repair process, and thus transitions occur more frequently than transversions (Mohanta and Bae, 2017). Our results of nucleotide sequence evolution analysis showed that the transition rate was higher than the transversion rate for the cotton genotypes evaluated, which is consistent with previous reports (Mohanta and Bae, 2017; Mohanta et al., 2019). SNP represents the most common form of polymorphism in biological genomes. Common polymorphisms are effective genetic markers related to biological evolution (Zhang et al., 2020). In the present study, we identified 4,074 SNPs in the *Gossypium* cp genomes. Among them, there were more SNPs in the intergenic region than the intron region, indicating that intergenic spacer sequences were more variable than intron regions in the plastid genome, which was consistent with the latest research results (Zhang et al., 2020). Furthermore, the dN/dS ratios were larger than 1, thereby indicating that non-synonymous mutations were fixed in the genomes, which may be due to component-driven mutation pressure (Foster et al., 1997). The dN/dS ratios were higher for the semi-wild and cultivated upland cotton genotypes than those determined for the wild cotton species, which may suggest that upland cotton has been subject to very strong artificial selection during domestication. The results of evolutionary rates indicated that the rates of nucleotide substitutions and indels were higher in wild species than the upland genotypes, thereby suggesting that the semi-wild and cultivated upland genotypes might have evolved more slowly after speciation. Due to the influence of artificial domestication, the cultivated genotypes exhibited less variation with fewer mutations. Previous studies have shown that selection can act on the mutation rate (Baer et al., 2007). Moreover, according to our results, the mutation rate was lower for indels than nucleotide substitutions, which is consistent with a previous report (Wu et al., 2018).

### Domestication Selection

By the mid-18th century, the coastal colonies of the southeastern United States had developed upland and Sea Island cotton varieties, which showed a long history of cotton domestication and breeding (Du et al., 2017). Evidence suggested that the domestication and breeding of allotetraploid cotton were superior to A-genomic diploid cotton in yield and quality (Hovav et al., 2008). And the allopolyploid cultivated cotton was first domesticated about 5,000 years ago (Yoo et al., 2014). Generally, synonymous and non-synonymous nucleotide substitutions are important markers of gene evolution. In most genes, synonymous nucleotide substitutions have occurred more frequently than non-synonymous substitutions (Ogawa et al., 1999). The rates of non-synonymous and synonymous substitutions are relatively slow in plant chloroplast genomes because of purifying and neutral selection (Erixon and Oxelman, 2008; Ivanova et al., 2017). In the present study, selection pressure analysis identified 16 genes with sites under positive selection in the wild species group, but only one of these genes (*rpl2*) was identified in the semi-wild and cultivated group. We conclude that the selection pressure on semi-wild and cultivated cotton species has fewer genes at positive selection sites, whereas the wild species retained adaptive genes and the selected sites increased. These results are generally consistent with those obtained in previous studies of the effects of artificial domestication on selection pressure (Price, 2002). When plants experience relatively large changes in the environment, such as artificial domestication or natural selection, the relaxation of selection for certain characteristics is inevitable (Coss, 1999; Price, 2002). Thereby, humans would expect that natural selection of these features would lose its strength (Price, 2002). The *rpl2* domestication selection gene identified in semi-wild and cultivated cotton species may have played an important role in the adaptation of *Gossypium* to various environments (Price, 2002; Fan et al., 2018; Wu et al., 2018; Chen et al., 2020). Moreover, selection pressure analysis for wild and domesticated cotton species can provide novel insights into how human selection has affected duplicated genes in allopolyploids (Yoo et al., 2014; Chen et al., 2020). It is known that many important crops such as potato, wheat and soybean are obvious polyploids, so studying the genes of allopolyploid cotton may provide new insights into the role of polyploids in crop evolution (Yoo et al., 2014).

### Genetic Diversity

Additionally, genetic diversity is the basis of crop improvement (Akter et al., 2019). Therefore, understanding the genetic diversity, structure, and relationships between varieties of upland cotton is very important for breeding (Fang et al., 2013). The semi-wild races exhibited higher nucleotide diversity (*H*_d_ = 1.000, π = 0.00035) than the cultivated genotypes (*H*_d_ = 0.946, π = 0.00010), thereby suggesting that artificial domestication reduced the chloroplast genetic diversity, which is consistent with a previous report (Ma et al., 2019). The low level of genetic diversity determined in the cultivated upland cotton accessions was primarily due to several genetic bottlenecks during the domestication process (Fang et al., 2013; Wang et al., 2017). Various studies have also suggested that the genetic basis of cultivated upland cotton genotypes is narrow (Abdurakhmonov et al., 2008; Campbell et al., 2009; Akter et al., 2019), although the diversity of derived cultivars obtained by various breeding methods is still evident. In addition, cotton breeding often involves hybridization and re-selection with a small number of breeding materials, thereby resulting in a loss of genetic diversity (Tyagi et al., 2014). The genetic structure is mainly affected by geographical isolation and genetic exchange isolation (Guo et al., 1997; Gutierrez et al., 2002). Genetic structure analysis showed that the semi-wild races and cultivated upland accessions were divided into two groups when *K* = 2. We observed that the seven semi-wild races and cultivated upland accessions exhibited significant admixture, that was, the two semi-wild races Marie-galante and latifolium had notable fractions assigned to cultivated accessions group, which indicated that the race latifolium had closest relationships with cultivated accessions, followed by the race marie-galante race, thereby indicating the introgression of a certain gene between the semi-wild races and cultivated accessions, or possibly germplasm sharing (Tyagi et al., 2014). These results were consistent with a previous study on increasing human-mediated effects leading to significantly genetic introgression (Du et al., 2017). A previous study also showed that the existence of this mixture may be related to the domestication history and the frequent appearance of superior genotypes in different breeding programs (Mulugeta et al., 2018). China is not a natural cotton-growing region, and thus many cotton genotypes, such as Foster, STV, DPL, Trice, King, and Uganda, have been introduced as extensive genetic sources for upland cotton varieties in China from several overseas sources for improving varieties (Chen and Du, 2006; Du et al., 2007; Jia et al., 2014a; Mulugeta et al., 2018). It is important to study the diversity and genetic structure of upland cotton genotypes as well as their relationships to facilitate the conservation and improvement of cotton (Mulugeta et al., 2018). In addition, the genetic diversity and population structure of upland cotton germplasm resources can be effectively used for genetic breeding, and it is of great significance for the systematic utilization of long-term genetic variation of upland cotton (Tyagi et al., 2014).

### Genetic Introgression

Ancient gene flow between domesticated varieties and their wild relatives probably occurred historically through seed transmission, and it was possibly influenced by human activities and environmental events (Wegier et al., 2011). In the present study, asymmetric historical gene flow was determined between the semi-wild and cultivated upland genotypes, which is consistent with a previous study (Deynze et al., 2011). However, contemporary gene flow was greatly reduced, which may have been due to current isolation. Genetic studies of species in the early stages of domestication have identified multiple domestication origins or high levels of sustained gene flow between wild and cultivated genotypes (Gross and Olsen, 2010). A previous study also suggested that the genetic structure of upland cotton genotypes was weak or an admixture, which may have resulted in a strong historical gene flow (Epps et al., 2013). In general, gene flow is an important factor that affects the population structure over time, where it may reduce local adaptation by homogenizing the populations found in different environments or by spreading harmful alleles between populations. Gene flow might also contribute to the introduction of potential adaptive alleles into populations and increased genetic variation (Sexton et al., 2011; Epps and Keyghobadi, 2015; Welt et al., 2015). Some studies have also indicated that gene flow from cultivated upland genotypes to wild cotton tetraploid species has increased the risk of extinction for these wild species (Wegier et al., 2011). I.e., some wild cotton species *G*. *tomentosum* (in Hawaii), *G*. *mustelinum* (in Brazil) and *G*. *darwinii* (in Galapagos) were in danger of extinction as a result of hybridization with domesticated tetraploid cotton (Ellstrand, 2003; Simard, 2010). In addition, numerous studies have shown that interspecific hybrids (*G*. *hirsutum* x *G*. *barbadense*) can serve as genetic links for gene transfer from domesticated cotton to other wild relatives (*G*. *darwinii*) (Ellstrand, 2003; Simard, 2010). This occurred during or after speciation lead to the retention of ancestral polymorphism due to incomplete lineage sorting (Heckman et al., 2007; Wilyard et al., 2009), or introgression or introgressive hybridization of previously geographically isolated species resulting from the genetic exchange after secondary contact (Liston et al., 1999; Gay et al., 2007). Moreover, among the four cultivated *Gossypium* plants, upland cotton exhibits the highest level of gene flow (Wendel et al., 1992; Abdurakhmonov et al., 2008), which is related to the strong artificial domestication that it has undergone. The extensive gene flow and/or genetic introgression among cotton accessions might have provided the novel genetic resources of cotton breeding. Therefore, the suitable management and conservation of different cotton species accessions are important in the future.

## Conclusion

In conclusion, our phylogenetic analysis confirms the evolutionary relationship within the whole *Gossypium*, especially the relationships between semi-wild races and cultivated accessions were well resolved. We also identified that the *rpl2* gene was positively selected in semi-wild races and cultivated genotypes. Meanwhile, we found that the cultivated genotypes have experienced very strong selection pressure. In addition, we found that the genetic diversity of cultivated accessions was low compared to wild ones due to artificial domestication. Through the analyses of genetic structure and gene flow, we concluded that there was a certain gene introgression between semi-wild races and cultivated accessions. The present research provided novel genetic resources for cotton breeding, as well as novel molecular mechanisms insights for the evolution and domestication of cotton species.

## Data Availability Statement

The original contributions presented in the study are included in the article/Supplementary Material, further inquiries can be directed to the corresponding author/s.

## Author Contributions

TZ, NW, and Z-HL: data curation and writing – original draft. YW: formal analysis. TZ, NW, and X-FM: investigation. YW, X-LZ, B-GL, WL, J-JS, C-XW, and AZ: methodology. X-FM: resources and validation. TZ and NW: software. Z-HL: supervision and writing – review & editing. All authors contributed to the article and approved the submitted version.

## Conflict of Interest

The authors declare that the research was conducted in the absence of any commercial or financial relationships that could be construed as a potential conflict of interest.

## Publisher’s Note

All claims expressed in this article are solely those of the authors and do not necessarily represent those of their affiliated organizations, or those of the publisher, the editors and the reviewers. Any product that may be evaluated in this article, or claim that may be made by its manufacturer, is not guaranteed or endorsed by the publisher.
