# Supplementary material for: Nucleotide Evolution, Domestication Selection, and Genetic Relationships of Chloroplast Genomes in the Economically Important Crop Genus Gossypium
Source: Front Plant Sci. 2022 Apr 15;13:873788. doi: 10.3389/fpls.2022.873788 (PMC9051515; doi:10.3389/fpls.2022.873788)
Supplement: Supplementary file 1 [file Data_Sheet_1.ZIP › Supplementary materials/Supplementary Material.docx]

Supplementary Material

# Supplementary Data

**Figure** **S1** Determination of the true number of genetic clusters in Bayesian clustering using the method described by Evanno et al. (2005). *ΔK* statistics are based on the rate of change in the log probability of data between successive *K* values.

**Table** **S1** Maximum likelihood parameter estimates for 77 genes in wild cotton species.

**Table** **S2** Likelihood ratio test (LRT) of the variable *ω* ratio under different models for wild species.

**Table S3** Maximum likelihood parameter estimates for 77 genes in cultivated cotton accessions.

**Table** **S4** Likelihood ratio test (LRT) of the variable *ω* ratio under different models for semi-wild and cultivated groups.

**Table S5** detailed information on nonsynonymous sites in Semi-wild and cultivated accessions of cotton.

**Table S6** detailed information on nonsynonymous sites in wild accessions of cotton.

## Supplementary Figures


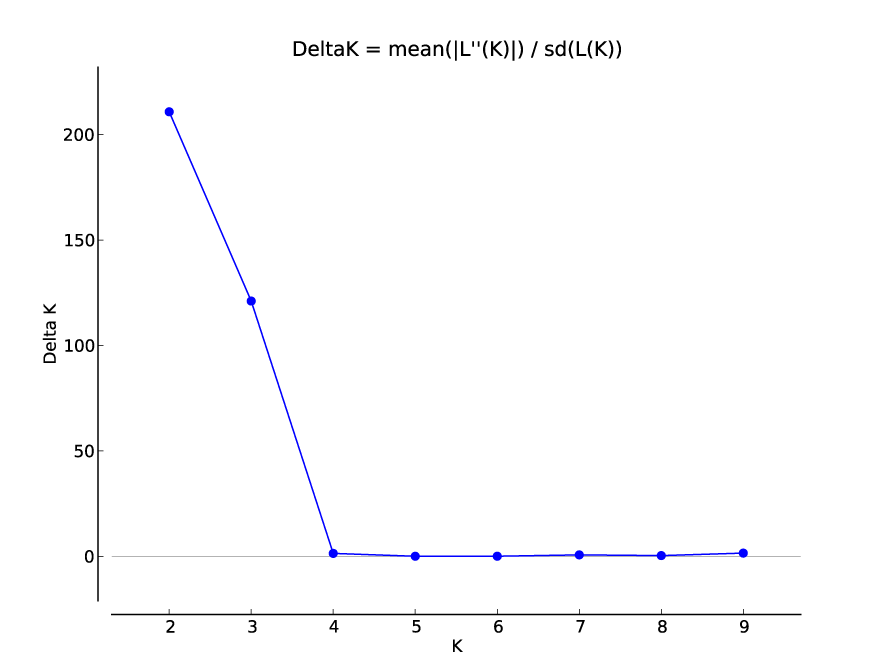


**Figure** **S1** Determination of the true number of genetic clusters in Bayesian clustering using the method described by Evanno et al. (2005). *ΔK* statistics are based on the rate of change in the log probability of data between successive *K* values.

## Supplementary Tables

Please see the Tables S1-S4.xlsx file for details.
