# Supplementary figures and images for: Nucleotide Evolution, Domestication Selection, and Genetic Relationships of Chloroplast Genomes in the Economically Important Crop Genus Gossypium
Source: Front Plant Sci. 2022 Apr 15;13:873788. doi: 10.3389/fpls.2022.873788 (PMC9051515; doi:10.3389/fpls.2022.873788)

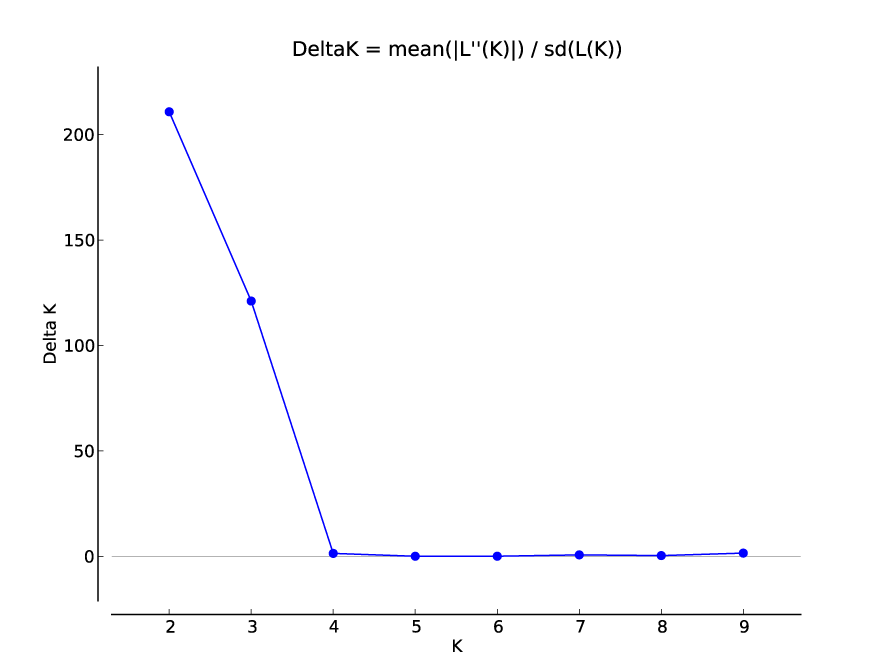

Supplement: Supplementary file 1 [file Data_Sheet_1.ZIP › Supplementary materials/Figure S1.tif]
